# An untargeted cultivation approach revealed *Pseudogemmatithrix spongiicola* gen. nov., sp. nov., and sheds light on the gemmatimonadotal mode of cell division: binary fission

**DOI:** 10.1038/s41598-024-67408-9

**Published:** 2024-07-21

**Authors:** Tom Haufschild, Nicolai Kallscheuer, Jonathan Hammer, Timo Kohn, Moses Kabuu, Mareike Jogler, Nicole Wohlfarth, Manfred Rohde, Muriel C. F. van Teeseling, Christian Jogler

**Affiliations:** 1 Department of Microbial Interactions, Institute of Microbiology, Friedrich Schiller University, Jena, Germany; 2 Leibniz Institute DSMZ, Brunswick, Germany; 3 Central Facility for Microscopy, Helmholtz Centre for Infection Research, Brunswick, Germany; 4 Cluster of Excellence Balance of the Microverse, Friedrich Schiller University, Jena, Germany

**Keywords:** Aquatic microbiology, Freshwater sponge, *Gemmatimonas*, Cell division, Binary fission, Budding, Cell division, Water microbiology

## Abstract

Members of the phylum *Gemmatimonadota* can account for up to 10% of the phylogenetic diversity in bacterial communities. However, a detailed investigation of their cell biology and ecological roles is restricted by currently only six characterized species. By combining low-nutrient media, empirically determined inoculation volumes and long incubation times in a 96-well plate cultivation platform, we isolated two strains from a limnic sponge that belong to this under-studied phylum. The characterization suggests that the two closely related strains constitute a novel species of a novel genus, for which we introduce the name *Pseudogemmatithrix spongiicola*. The here demonstrated isolation of novel members from an under-studied bacterial phylum substantiates that the cultivation platform can provide access to axenic bacterial cultures from various environmental samples. Similar to previously described members of the phylum, the novel isolates form spherical appendages at the cell poles that were believed to be daughter cells resulting from asymmetric cell division by budding. However, time-lapse microscopy experiments and quantitative image analysis showed that the spherical appendages never grew or divided. Although the role of these spherical cells remains enigmatic, our data suggests that cells of the phylum *Gemmatimonadota* divide via FtsZ-based binary fission with different division plane localization patterns than in other bacterial phyla.

## Introduction

Bacteria are remarkably diverse with respect to their phylogeny, metabolic potential, ecological niche adaptation and their cell biology, such as, cell shape or cell division. The majority of this diversity remains undiscovered, as it is estimated that only 2% of all bacteria have been cultivated^1^. Our picture of diversity thus comes from this small fraction of bacterial strains, often fast-growing and easily genetically manipulable—the ‘known knowns’. While powerful metagenomic approaches point towards the remaining 98%—the ‘known unknowns’, we are not even close to predict for example their cell shape, mode of cell division or other novel cell biological features solely based on genomic information.

Furthermore, others and we cultivated bacteria that were initially missed by cultivation-independent techniques before^2–8^. Obtaining novel axenic bacterial cultures remains indispensable for both, in-depth characterization of the ‘known unknowns’ and the future discovery of the ‘unknown unknowns’ of which some can introduce major paradigm shifts^9^.

The cultivation and isolation of slow-growing strains is labour-intensive as it is based on advanced cultivation techniques and longer incubation times. Here, we applied a less labour-intensive untargeted cultivation approach that relies on the seeding of diluted environmental samples into 96-well plates such that one viable cell is present per well and can grow into an axenic culture. In contrast to classical agar plate cultivation, the system is easily scalable and automatable and can capture bacteria that do not grow on agar plates. 

One example of understudied bacteria are members of the phylum *Gemmatimonadota*. Although they were shown to account for 2–10% of the bacterial community according to cultivation-independent 16S rRNA gene amplicon sequencing studies^10,11^, a detailed investigation of their cell biology and ecological roles is restricted by the low number of characterized isolates. The first characterized member of the phylum, *Gemmatimonas aurantiaca* T-27^T^, was isolated from a wastewater batch reactor in the context of a study targeting the identification of polyphosphate-accumulating bacteria^12^. The approach combined low-nutrient cultivation media, prolonged incubation times, and mild harvesting conditions (low-speed centrifugation). With the description of two additional members of the genus *Gemmatimonas* in the last decade^13,14^, this genus is currently the only one in the phylum with more than one described representative. The three *Gemmatimonas* species, together with *Gemmatirosa kalamazoonensis* KBS 708^T^, *Roseisolibacter agri* AW1220^T^, and the more distantly related *Longimicrobium terrae* CB 286315^T^, constitute the current phylum *Gemmatimonadota*^15–17^. Uncommon bacterial characteristics have been observed in the described members of the phylum, which include the formation of spherical appendages. This eponymous feature (*Gemmatimonas*: “a budding unit”) was observed in all members of the phylum except *L. terrae* and was suggested to be the result of cell division by budding in addition to cell division by binary fission^13–15,17^. The term “budding” describes various forms of asymmetric cell division by which a daughter cell emerges at one of the cell poles, grows and eventually pinches off from the mother cell. So far, budding has been observed in various bacterial strains, each with their own curiosities^18^. Such strains belong to species from the phyla *Pseudomonadota* (especially within the *Alphaproteobacteria*^19^), *Planctomycetota* (class *Planctomycetia*^3^), and *Gemmatimonadota*^12^.

Physiological analyses showed that the type strains of *Gemmatimonas phototrophica* and *Gemmatimonas groenlandica* are facultative photoheterotrophs that acquired photosynthesis-related genes via horizontal gene transfer, likely from a purple phototrophic bacterium^13,14^. The phototrophic lifestyle is associated with characteristic photosynthetic reaction centers and the biosynthesis of carotenoids designated gemmatoxanthins^20,21^. An analysis of biomarker genes suggested that phototrophic Gemmatimonadetes bacteria (PGB) are widely distributed in aquatic and terrestrial environments.

In this study, we sampled material from a sponge specimen in an artificially created lake in Northern Germany. The subsequent untargeted cultivation in 96-well plate format with defined inoculation amounts and prolonged incubation time yielded two closely related strains (Strain 318^T^ and Strain 138) that belong to the phylum *Gemmatimonadota*. An in-depth characterization of the cell biology of these isolates using time-lapse microscopy and quantitative image analysis revealed that under laboratory conditions the gemmatimonadotal structures previously described as buds are no viable daughter cells and that cells instead divide via binary fission. These results thus suggest that reproduction by budding occurs in less bacterial phyla than is currently assumed. Furthermore, our study demonstrates that the untargeted cultivation pipeline is suitable to isolate elusive microbes and can be scaled up to high-throughput cultivation more easily than classical agar plate cultivation.

## Material and methods

### Sampling and strain isolation

**Figure 1:**
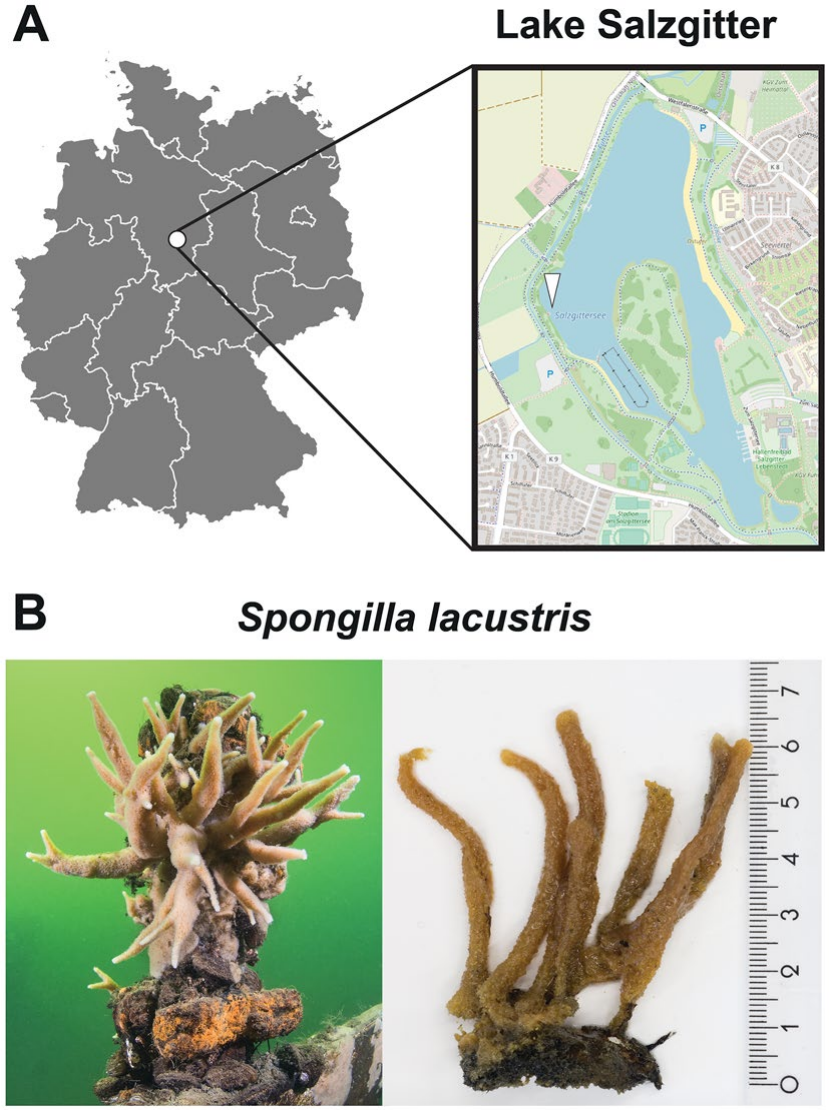
Sampling location and sampled sponge specimen. (**A**) Lake Salzgitter in Northern Germany was sampled during a diving expedition. The white arrowhead indicates the exact sampling site. (**B**) Material from the depicted sponge specimen was used for the isolation of Strain 318^T^ and Strain 138. The map showing Lake Salzgitter was obtained from OpenStreetMap (CC-BY-SA 2.0, https://openstreetmap.de, accessed on the 10th of January 2024).

In our quest to bring uncharacterized strains into axenic culture, we took samples during a scientific diving expedition in September 2016. During this expedition, a specimen of the freshwater sponge *Spongilla lacustris* was collected from Lake Salzgitter in Northern Germany (longitude: 52.156695, latitude: 10.298833, Fig. 1). The sponge specimen was stored at 4 °C in water obtained from Lake Salzgitter sterilized through a 0.2 µm filter (Millipore GmbH). Loosely associated bacteria were washed out by exchanging the water surrounding the sponge three times with novel batches of sterilized tap water. The washed sponge tissue was disrupted by repeated pipetting with a sterile serological pipette. The resulting suspension was filtered through a 10 µm pore filter to remove residual sponge fragments and cells. To determine bacterial cell numbers in the filtered fraction, a fraction was fixed in 2% (v/v) glutaraldehyde for 10 min and subsequently stained with the DNA dye 4′,6-diamidino-2-phenylindole (DAPI). Stained bacterial cells were collected on black polycarbonate filters with a pore size of 0.2 μm (Millipore GmbH). The quantification proceeded via epifluorescence microscopy.

Based on the cell counts, wells from a round-bottom 96-well plate containing 200 µL of V2mod medium were stochastically inoculated with either 50 or 200 cells per well using a multidrop combi apparatus (Thermo Electron Corporation, Finland). V2mod medium consisted of 2.38 g/L HEPES (4-(2-hydroxyethyl)-1-piperazineethanesulfonic acid), 0.53 mg/L NH_4_Cl, 1.4 mg/L KH_2_PO_4_, 10 mg/L KNO_3_, 49.3 mg/L MgSO_4_ × 7 H_2_O, 14.7 mg/L CaCl_2_ × 2 H_2_O, 25 mg/L CaCO_3_, 25 mg/L NaHCO_3_, 20 mL Hutner’s basal salt solution, 0.1 g/L Bacto peptone and 0.1 g/L Bacto yeast extract. The pH was adjusted to 7.2 with 5 M KOH. After autoclavation of the medium (20 min at 121 °C) and cooling, the following sterile-filtered solutions were added: 4 mL of 2.5% (w/v) glucose (final concentration 0.1 g/L), 2 mL of 5% (w/v) *N-*acetyl glucosamine (NAG) (final concentration 0.1 g/L), 5 mL vitamin solution, 1 mL trace element solution and 100 mg/L cycloheximide (to inhibit fungal growth). Hutner’s basal salt solution contained (in g/L): nitrilotriacetic acid, 10; MgSO_4_ × 7 H_2_O, 30; CaCl_2_ × 2 H_2_O, 3.5; (NH_4_)_6_MoO_7_O_24_ × 4 H_2_O, 0.01; FeSO_4_ × 7 H_2_O, 0.1; and metal stock solution, 50 mL. The metal stock solution contained (in g/L): Na-EDTA, 0.25; ZnSO_4_ × 7 H_2_O, 1.1; FeSO_4_ × 7 H_2_O, 0.5; MnSO_4_ x H_2_O, 0.15; CuSO_4_ × 5 H_2_O, 0.04; Co(NO_3_)_2_ × 6 H_2_O, 0.025; Na_2_B_4_O_7_ × 10 H_2_O, 0.018. The vitamin solution contained (in mg/L): cyanocobalamin (vitamin B_12_), 4; biotin, 4; thiamine-HCl × 2 H_2_O, 10; Calcium pantothenate, 10; folic acid, 4; riboflavin, 10; nicotinamide, 10; *p*-aminobenzoic acid, 10; pyridoxine × HCl, 20. The trace element solution contained (in mg/L): Na-nitrilotriacetate, 1500; MnSO_4_ × H_2_O, 500; FeSO_4_ × 7 H_2_O, 100; Co(NO_3_)_2_ × 6 H_2_O, 100; ZnCl_2_, 100; NiCl_2_ × 6 H_2_O, 50; H_2_SeO_3_, 50; CuSO_4_ × 5 H_2_O, 10; AlK(SO_4_)_2_ × 12 H_2_O, 10; H_3_BO_3_, 10; NaMoO_4_ × 2 H_2_O, 10; and Na_2_WO_4_ × 2 H_2_O, 10. All solutions that were added after the autoclavation step were filter-sterilized and stored at 4 °C in the dark. For solidified medium 15 g/L agar (washed three times with double-distilled H_2_O) was autoclaved separately and added prior to pouring of the plates.

The 96-well plate inoculated with bacterial cells obtained from the sponge was incubated at 20 °C and growth was followed on a weekly basis by visual inspection of turbidity and colour of the contents of each well. Wells showing growth after up to 9 months of incubation were subjected to PCR-based amplification of the 16S rRNA gene based on a previously published protocol^22^ to identify the bacterial isolate and to check for purity. Cultivation of strains of interest as determined by their 16S rRNA gene sequence proceeded by first scaling up the sample volume in 96-well plates by adding V2mod medium and further incubating the samples at 20 °C. Then, the culture volume was increased stepwise and, when appropriate, the cultures were transferred from 96-well plates to cultivation flasks that were incubated under gentle agitation (70 rpm) at 28 °C in limnic M3 medium. Limnic M3 medium was prepared based on the recipe of V2mod medium with the following alterations: 1 g/L Bacto peptone, 1 g/L Bacto yeast extract, the pH was adjusted to 7.0 with 5 M KOH, 4 mL 25% (w/v) glucose solution (final concentration 1 g/L), 20 mL 5% (w/v) *N*-acetylglucosamine (final concentration 1 g/L).

### DNA extraction, genome sequencing, and genome assembly

DNA extraction, sequencing, and genome assembly were performed according to a previously published workflow^23^.

### Nucleotide sequence accession numbers

The 16S rRNA gene sequence of the two isolates is available from GenBank under the accession numbers OR304275 (Strain 138) and OR336385 (Strain 318^T^). The genome sequence can be found under the accession numbers CP130612 (Strain 138) and CP130613 (Strain 318^T^).

### Phylogenetic analyses

The 16S rRNA gene sequences of the two isolated strains were extracted from the annotated genomes and the identification of their closest neighbours was performed using NCBI BLASTn^24^. The 16S rRNA gene sequence of the novel strains and all characterized members of the current phylum *Gemmatimonadota* were aligned with ClustalW^25^. The alignment was used to calculate a sequence similarity matrix with TaxonDC^26^. A maximum likelihood phylogenetic tree was calculated from the same alignment with FastTree employing the GTR + CAT model and 1000 bootstrap replications^27^. Three 16S rRNA genes of bacterial strains from the phylum *Planctomycetota*, namely *Rhodopirellula baltica* SH1^T^ (NCBI acc. no. NR_043384), *Planctopirus limnophila* DSM 3776^T^ (acc. no. NR_029225), and *Isosphaera pallida* IS1B^T^ (acc. no. NR_028892), were used as outgroup. The multi-locus sequence analysis (MLSA)-based phylogenetic inference was performed using autoMLST with 1000 bootstrap replicates^28^. The analysis included all reference genomes of strains belonging to the current phylum *Gemmatimonadota*. The genomes of *R. baltica* SH1^T^ (GenBank acc. no GCA_000196115.1),* P. limnophila* DSM 3776^T^ (acc. no. GCA_000092105.1), and* I. pallida* IS1B^T^ (acc. no. GCA_000186345.1) served as outgroup. Phylogenetic trees were visualized with iTOL v6^29^. Average nucleotide identities (ANI) and average amino acid identities (AAI) were obtained using the respective scripts of the enveomics collection^30^. The percentage of conserved proteins (POCP) was calculated as described^31^.

### Analysis of genome-encoded features

The genomes of the novel isolates and the reference genomes of the current members of the phylum *Gemmatimonadota* were re-annotated using NCBI’s PGAP pipeline (version 2023-05-17.build6771). This step was included to ensure that the obtained data is comparable and that proteins are annotated based on the most recent annotation data. The pangenome of all strains was constructed with the pangenomics workflow of anvi’o v. 7.1^32^. The “Estimate Metabolism” function of the same tool was used for the analysis of genome-encoded primary metabolic functions. Numbers of putative carbohydrate-active enzymes (CAZymes) were obtained from the genome annotation provided by eggnog-mapper v.2.1.10^33^. An in silico prediction of biosynthetic gene clusters (BGCs) putatively involved in the biosynthesis of secondary metabolites was carried out using antiSMASH 7.0.1^34^. The prediction was run with relaxed strictness and all extra features activated. The genome completeness was assessed with BUSCO v5.4.7^35^, while the coding density was analysed with CheckM v1.1.6^36^.

### Physiological analyses

For determination of the temperature optimum for growth, 150 µL of a 1:1000 diluted exponentially growing culture were streaked on limnic M3 medium agar plates in biological duplicates and cultivated for eight weeks at different temperatures ranging from 4 to 42 °C. The plates were regularly inspected for growth and the optimal temperature for growth was determined visually. The pH range for growth was tested at the determined optimal growth temperature in liquid limnic M3 medium using different buffer systems. A concentration of 100 mM HEPES was used for cultivations at pH 7.0, 7.5 and 8.0. For the cultivation at pH 5.0 and 6.0 HEPES was replaced by 100 mM 2-(*N*-morpholino)ethanesulfonic acid (MES), whereas 100 mM *N*-cyclohexyl-2-aminoethane-sulfonic acid (CHES) served as a buffering agent at pH 9.0 and 10.0. Vitamin requirements of the isolates were tested in limnic M3 medium either without supplemented vitamins, with only vitamin B_12_ (final concentration 20 µg/L) or with a vitamin solution containing (final concentrations in brackets) *p*-aminobenzoate (50 µg/L), biotin (20 µg/L), pyridoxine hydrochloride (100 µg/L), thiamine hydrochloride (50 µg/L), calcium pantothenate (50 µg/L), folic acid (20 µg/L), riboflavin (50 µg/L), nicotinamide (50 µg/L) and vitamin B_12_ (20 µg/L). Cultivations for determination of the pH optimum and vitamin requirements were performed in two biological replicates, each in technical duplicates, in an Epoch2 microplate spectrophotometer (Agilent, Waldbronn, Germany). The microtiter plate wells were inoculated to an initial optimal density at 600 nm (OD_600_) of 0.05.

### Liquid–liquid two phase extraction and antimicrobial activity assay

For testing potential antimicrobial activity of compounds produced by the type strain (Strain 318^ T^), a liquid–liquid two phase extract of the culture supernatant was prepared. The strain was cultivated in 120 mL limnic M3 medium for nine weeks at 28 °C. After harvesting of the cells by centrifugation (4350 × g, 15 min, room temperature), the supernatant was filtered through a 0.2 µm filter (Millipore) and acidified to pH 4.0 with 1 M HCl. An equal volume of ethyl acetate was added, and the mixture was shaken for 30 min at 180 rpm. Subsequently, the organic phase was separated from the aqueous phase using a separation funnel and the solvent (ethyl acetate) was evaporated in a rotary evaporator (Rotavapor R-114 connected to a B-480 water bath, Büchi) at 40 °C and 240 mbar. The extract was evaporated to dryness and resuspended in 200 µL methanol. Subsequently, 20 µL of the resuspended extract was added to a filter disk (6 mm) and allowed to dry for ca. 20 min. The disks were placed on LB plates inoculated with the indicator strains *Escherichia coli* ∆*tolC*^37^ (a mutant lacking TolC that is part of efflux pumps for toxic compounds) and *Bacillus subtilis* DSM10^T^. The plates were incubated at 28 °C. Discs with kanamycin (10 µg per disc) were used as a positive control while 20 µL methanol and 20 µL sterile double-distilled H_2_O served as negative controls. The plates were inspected for zones of inhibition after 24 h of incubation.

### Light and electron microscopy

Cell morphological features and cell division were analysed by light microscopy and field emission scanning electron microscopy (FESEM). Phase contrast (PhC) images were obtained using a Nikon Eclipse Ti2 inverted microscope equipped with a Nikon Plan Apo λ 100 × immersion oil objective (with phase ring for PhC), a Nikon Plan Apo λ 100 × immersion oil objective (without phase ring for differential interference contrast, DIC), a Nikon DS-Ri2 camera, and the NIS-Elements software (version 5.30). Both strains were grown in limnic M3 medium in technical triplicates until the mid-exponential phase (maximum OD_600_: 0.8). On the imaging day, the OD_600_ of the culture was determined. Two microliters of the culture were placed on a 1% (w/v) agarose cushion (prepared with autoclaved water) and were imaged with the phase contast (PhC) and differential interference contract (DIC) set-up. For time-lapse microscopy, Strain 318^T^ was grown until an OD_600_ of 0.3. Two microliters of culture were placed on a 1% (w/v) agarose cushion (prepared with limnic M3 medium). The cover slip was sealed with VLAP (33% vaseline, 33% lanoline, 33% paraffin) to prevent evaporation during imaging. Cells were imaged with the PhC set-up and a 2 × magnification lens. Images were taken every 40 min over a duration of 120 h at multiple locations. For DAPI and Synaptored staining, 500 µL of a mid-exponential culture of Strain 318^T^ was mixed with 3 µL DAPI (500 µg/mL) and 1 µL Synaptored (1 mg/mL) and incubated for 30 min. Cells were washed three times with limnic M3 medium and two microliters were applied on a 1% (w/v) agarose cushion (agarose diluted in water). Phase contrast and fluorescence images were taken with the same microscope set-up as described before, but using an Orca-flash 4.0 camera for image acquisition, a 1× magnification lens, a DAPI filter (Semrock; DAPI-1160B) and a Texas Red longpass filter (AHF; 560/40x, 600 DC, 610 LP). Cells were counted manually using the multi-tool in FIJI on nine fields of view. For visualization, the three-channel images were transformed into RGB images. If necessary, the contrast was enhanced for proper visualization. Movies were stabilized with the Image Stabilizer Plug-In^38^ for FIJI and scale bar/time stamps were added to images and movies with FIJI.

For FESEM, cells in the exponential phase were fixed in 1% (v/v) formaldehyde in HEPES buffer (3 mM HEPES, 0.3 mM CaCl_2_, 0.3 mM MgCl_2_, 2.7 mM sucrose, pH 6.9) for 1 h on ice and washed with the same buffer. A volume of 50 µL of the fixed bacteria solution was placed on a poly-l-lysine-coated cover slip and allowed to settle for 10 min. Cover slips were then fixed in 1% (v/v) glutaraldehyde in TE buffer (20 mM TRIS, 1 mM EDTA, pH 6.9) for 5 min. at room temperature and subsequently washed twice with TE buffer before dehydrating in a graded series of acetone (10, 30, 50, 70, 90, 100%, (v/v)) on ice for 10 min at each concentration. Samples from the 100% acetone step were brought to room temperature before placing them in fresh 100% acetone. Samples were then subjected to critical point drying with liquid CO_2_ (CPD 300, Leica). Dried samples were covered with a gold/palladium (80/20) film by sputter coating (SCD 500, Bal-Tec) before examination in a field emission scanning electron microscope (Zeiss Merlin) using the Everhart Thornley HESE2 detector and the inlens SE detector in a 25:75 ratio at an acceleration voltage of 5 kV.

### Cell size determination and cell division site localization

Images from the DS-Ri2 camera were processed in FIJI (Version 2.9.0)^39^ for further use in BacStalk (Version 1.8)^40^. For this purpose, the three-channel images were transformed into RGB images and saved as tiff files. If necessary and only for proper visualization in figures, the contrast was slightly enhanced. In BacStalk, images of one strain and one replicate were loaded at a time and the pixel size was adjusted to 0.029 µm. The cell length and width of 150 cells per replicate were determined automatically, the correct selection of cells was checked manually. For visualization, the data was uploaded to SuperPlotsofData^41^. Images for the visualization of (a)symmetrically dividing cells and division site localization analyses were processed in FIJI as described above. The length from either cell pole to the division site was then measured with the in-built measurement tool in FIJI. If one cell was too long for one single measurement, multiple measurements were taken and added up. Different time points were used to identify division sites and a total of 300 cells from various imaging locations were analysed. The total cell length (defined as cell length of the mother cell) was calculated by adding up the distances of the division plane to both cell poles. The cell length of the daughter cell was defined as the smaller of both length measurements. To determine the relative position of the division site (*S*), the cell length of the daughter cell was divided by the cell length of the mother cell. Since it was not possible to determine a cell pole, the data was mirrored in order to represent the possibility of the division on the other pole (indicated with a dashed line in the figures). The linear functions visualized in Fig. 7C have a slope based on *S* calculated for the respective groups. Data points were grouped by visual inspection in terms of cell length and relative division site as shown in Fig. 7F, mean values were calculated from data points assigned to those groups. For visualization of the relative division site *S* (Fig. 7D and E), the data points of the same 300 cells were used and mirrored on the dashed line. For data plotting RStudio (Version 2022.07.1 + 554)^42^ was used with the ggplot2 package^43^.

### Ethical approval

This article does not contain any studies with animals performed by any of the authors.

## Results and discussion

Our untargeted cultivation approach, starting from freshwater sponge samples, resulted in 48 strains that were considered slow-growing by regular manual inspection. Such strains were potentially novel and 16S rRNA gene sequencing revealed that two of them (Strain 138 and Strain 318^T^) belong to the yet understudied phylum *Gemmatimonadota*. Both were selected for characterization.

### Phylogenetic analyses delineate the novel isolates from previously characterized genera

**Figure 2:**
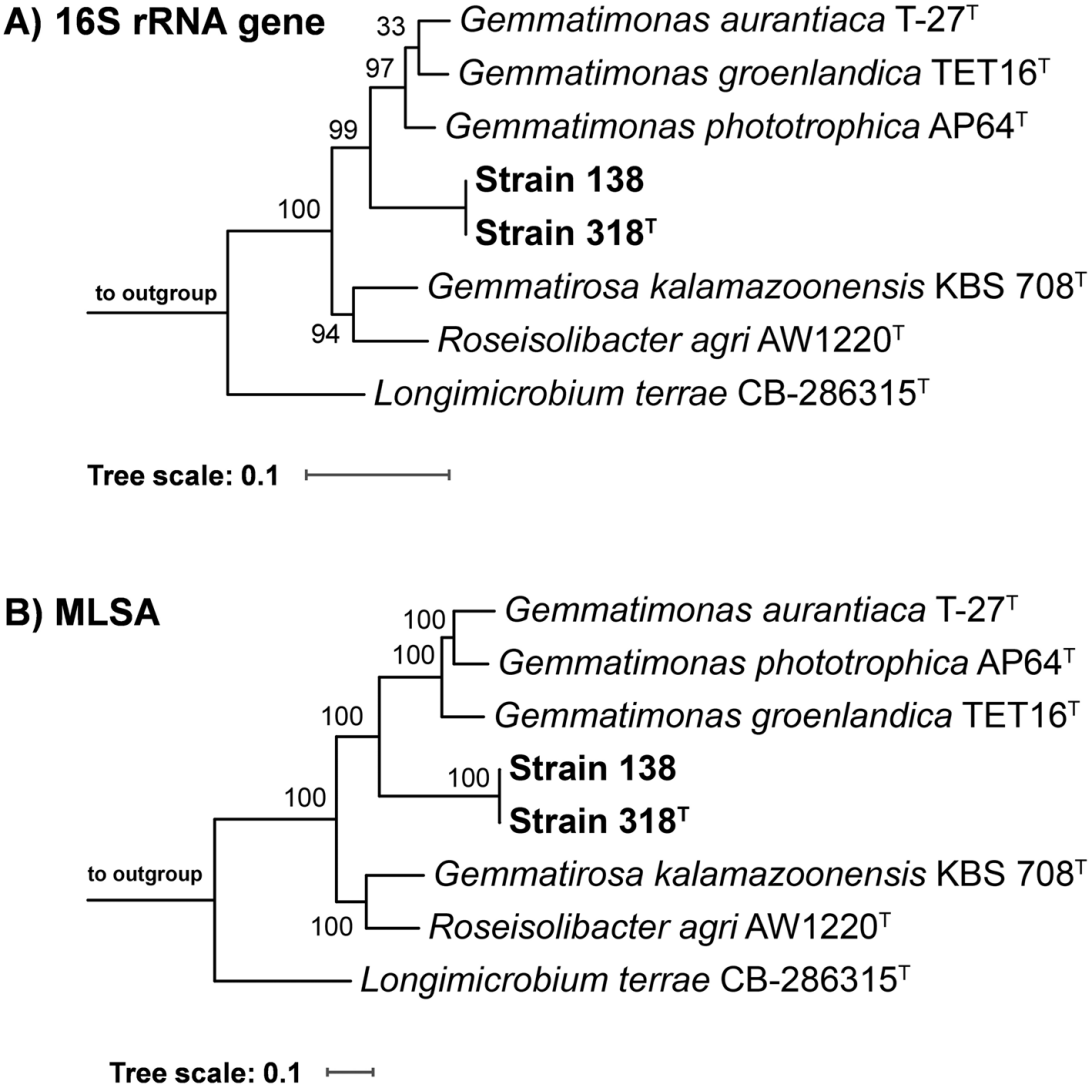
Maximum likelihood phylogenetic trees showing the position of the novel isolates. (**A**) 16S rRNA gene sequence- and (**B**) MLSA-based phylogenies were computed based on the current members of the phylum *Gemmatimonadota*. Bootstrap values after 1000 re-sampling are given at the nodes (in %). Phylogenetic trees were visualized with iTOL v6. The scale bar indicates the number of substitutions per nucleotide position.

**Figure 3:**
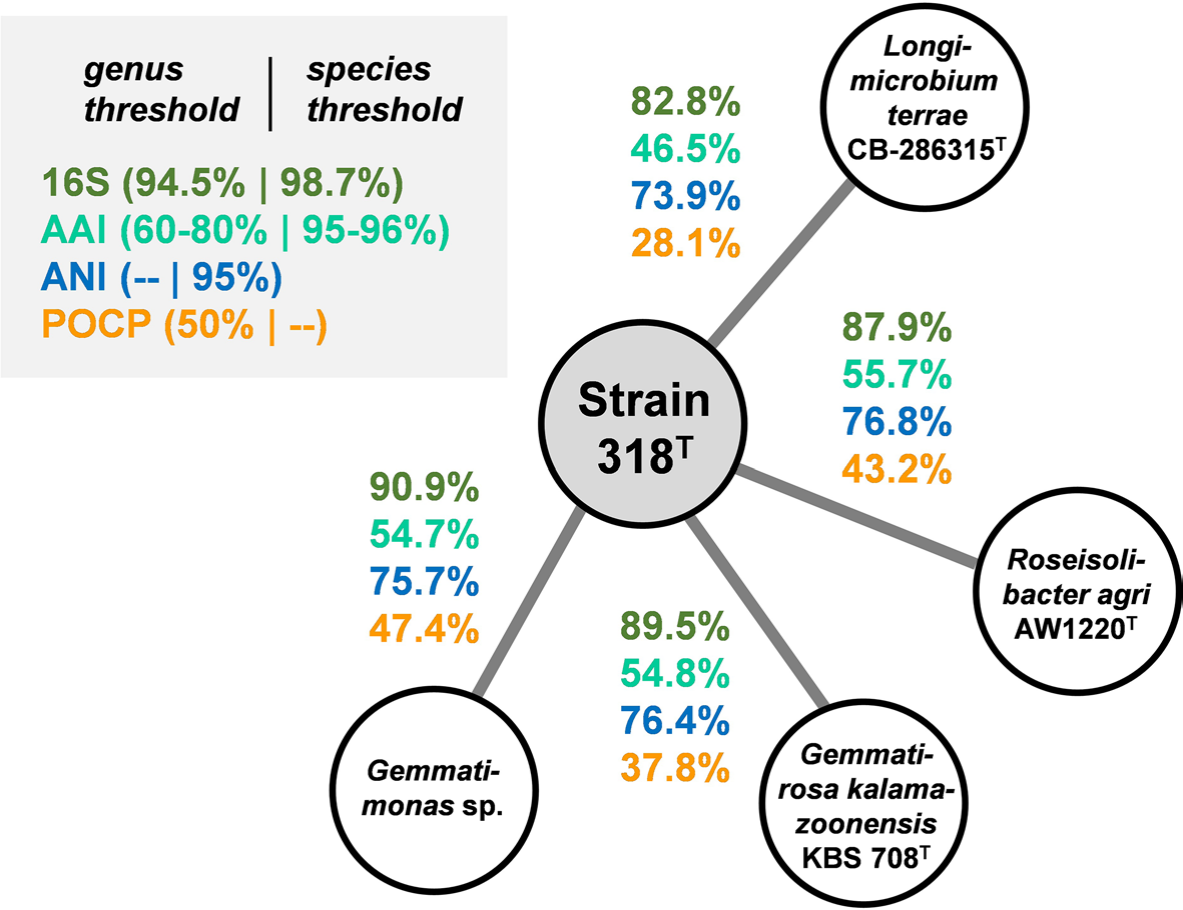
Analysis of phylogenetic markers shows that the novel isolates together belong to a novel species of a novel genus. Similarity values shared between Strain 318^T^ and the type strains of the current closest related species are depicted. The analysed phylogenetic markers included 16S rRNA gene sequence similarity, average amino acid identity (AAI), average nucleotide identity (ANI) and percentage of conserved proteins (POCP).

To identify the current closest relatives of Strain 138 and Strain 318^T^, their identical 1521 bp 16S rRNA gene sequence was used for a BLASTn analysis against the rRNA/ITS database of NCBI. The analysis yielded the three characterized members of the genus *Gemmatimonas*, *G.* *aurantiaca* T-27^T^^12^, *G.* *phototrophica* AP64^T^^14^, and *G. groenlandica* TET16^T^^13^, as closest relatives. The 16S rRNA gene sequence similarity to the three type strains turned out to fall between 90.9 and 91.7%, which places the newly isolated strains in the phylum *Gemmatimonadota*^44^. This phylum is barely explored and only six members have been characterized so far. These include the three above-mentioned *Gemmatimonas* species as well as *R. agri*^15^, *G. kalamazoonensis*^17^ and the more distantly related *L. terrae*^16^. In the constructed 16S rRNA gene sequence-based maximum likelihood phylogenetic tree (Fig. 2A), the novel isolates cluster next to the *Gemmatimonas* species, which is in line with the BLASTn-based sequence similarities. The MLSA-based tree showed the same clustering pattern as the 16S rRNA gene sequence-based tree (Fig. 2B), but with better reliability (100% bootstrap support on all branches). To analyse the phylogenetic position of the novel isolates in more detail, two-way comparisons of all type strains were performed for different established phylogenetic markers including 16S rRNA gene sequence similarity, ANI, AAI and POCP (Fig. 3 and Tables S1-S4). The 16S rRNA gene sequence similarity matrix gave the above-mentioned highest similarity value of 91.7% during the comparison of Strain 138/Strain 318^T^ with the type strain of *G. phototrophica*. This value falls below the proposed threshold of 94.5% used for the delineation of genera^45^, suggesting that the novel isolates belong to a novel species of a separate genus. The similarity matrix also showed that, except for the type strain of *L. terrae*, similarity values for all strain combinations fall above the family threshold of 86.5%^45^. When *L. terrae* is also considered, the lowest obtained similarity value is 82.8% (obtained for the comparison with the novel isolates Strain 138 and Strain 318^T^). This value is slightly above the proposed order threshold of 82.0%^45^. The obtained values are in line with the current placement of all species in the family *Gemmatimonadaceae* except *L. terrae*. When following the order threshold strictly, the family *Longimicrobiaceae* (currently harbouring *Longimicrobium* as the sole genus) should be transferred to the order *Gemmatimonadales* instead of assigning it to the separate order *Longimicrobiales* of the separate class *Longimicrobiia*.

For Strain 138 and Strain 318^T^, the genome-based phylogenetic markers support the assignment of the two closely related strains to a single novel species belonging to a genus that is delineated from the current closest related genus *Gemmatimonas*. For AAI and POCP, the obtained values for the novel isolates obtained during the two-way comparison of all type strains fall below the proposed genus thresholds of 60–80% for AAI and 50% for POCP^31,46^. Maximum ANI values of 76.8% ensure that the novel isolates do not belong to any characterized species (95% threshold value). The very close relationship between the two isolates is reflected by identical 16S rRNA gene sequences and ANI, AAI and POCP values above 99.8%. Hence, both belong to the same species.

### The two novel isolates possess reduced genomes

Besides phylogenetic analyses, the two obtained genomes were used for more detailed comparisons to the other phylum members. Prior to comparing genotypic features of the six characterized members of the phylum *Gemmatimonadota* and the two novel isolates, all genomes were re-annotated with the same version of the NCBI PGAP pipeline. This step was included to ensure that the obtained numbers of protein-coding genes are comparable, and the coding genes are annotated based on the most recent version of the tool. As expected from the high ANI, AAI and POCP values obtained during comparison of the two novel isolates, they are very closely related. The genome of Strain 138 is 2580 bp larger than the genome of Strain 318^T^. This difference is caused by the presence of two additional adjacent genes in Strain 138 that code for a hypothetical protein and a putative TIR domain-containing protein. Both genes are encoded in a prophage region with putative integrase- and transposase-encoding genes in the neighbouring regions upstream and downstream, respectively. Since none of the two isolates showed a distinct phenotype, single nucleotide polymorphisms in other protein-coding genes were not analysed at this stage.

The most striking difference among the current *Gemmatimonadota* genomes is their size. With 3.3 Mb, the novel isolates have much smaller genomes compared to the other members of the phylum, for which genome sizes of 4.6–7.5 Mb have been determined. All members have a high DNA G + C content (64–74%) and with 69% the novel isolates are close to the average. The reduced genome size of the novel isolates goes along with a higher coding density (93% vs. 90–92%) and a higher number of genes per Mb (911 vs. 830–860). The type strain of *L. terrae* stood out with a much lower coding density of 83.1%, which might reflect the greater phylogenetic distance of this strain. Plasmids were not observed in *Gemmatimonas* spp. and the novel isolates. The type strain of *G. kalamazoonensis* harbours a putative phage-like extrachromosomal element, for the strains with an incomplete genome sequence the information on the lack of extrachromosomal elements remains tentative. Numbers of tRNAs (46–64) and rRNAs (1–2 each for 5S, 16S, and 23S rRNA genes) are similar among the compared strains. The same is true for the relative number of hypothetical protein-encoding genes (20–30%), except for *R. agri* AW1220^T^ (NCBI Bioproject acc. no. PRJDB14149), for which 61% of the protein-coding genes are annotated as hypothetical proteins. However, this might be related to the low sequence quality of the draft genome. The related strain *Roseisolibacter* sp. H3M3-2 (GenBank acc. no. GCA_029211165.1), which is not validly described, yielded a comparable relative number of 28% hypothetical proteins for a slightly smaller genome (5.4 Mb).

**Figure 4:**
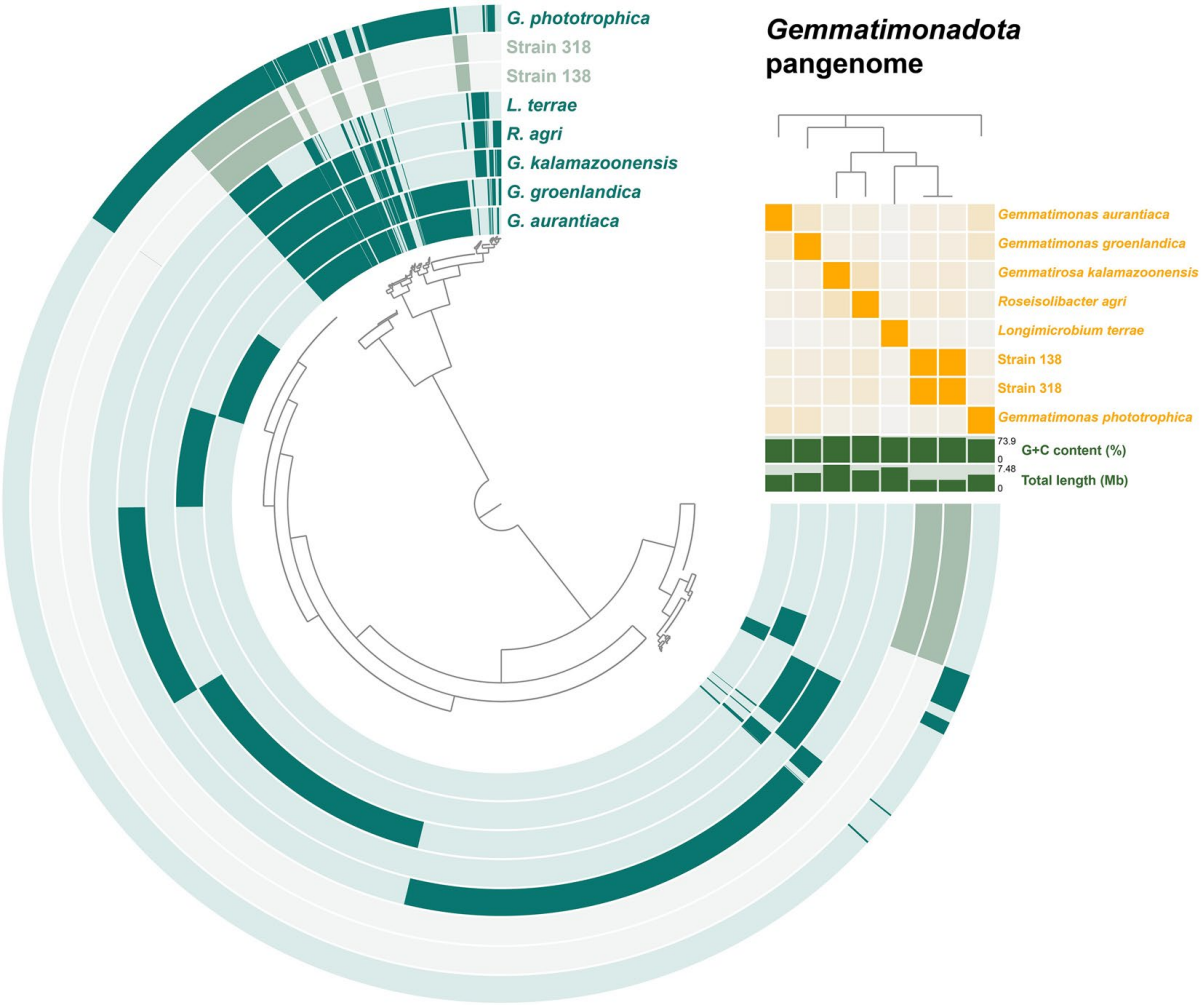
Pangenome of all current members of the phylum *Gemmatimonadota*. Each open circle represents the pangenome of all strains but is coloured darker when the gene is present in the respective genome. The matrix in the upper right corner indicates the degree of relationship of the strains according to average nucleotide identity values.

To visualize the degree of genomic relationship, a pangenome was constructed based on the eight available genomes (Fig. 4). The analysis yielded a total number of 19,205 genes for the pangenome, of which 484 were found in all eight genomes and 920 in all genomes except *L. terrae* CB-286315^T^. The pangenome was also used to compare Strain 318^T^ and Strain 138, which provided the information on the two additional genes present in Strain 138. When leaving out the genome of Strain 138 (due to redundancy with Strain 318^T^), 80% of the genes of Strain 318^T^ were identified as singleton genes. The highest number of singletons (4345 genes) could be attributed to the type strain of *L. terrae*. This is not surprising as this strain is most distantly related and has the second largest genome.

### Bioinformatic analyses of the novel type strain suggest a facultatively photoheterotrophic lifestyle but no extensive secondary metabolome

With the genomes of the novel isolates in hand, we performed bioinformatic analyses to get insights into the metabolic potential of the novel genus. Therefore, the “Estimate Metabolism” function of anvi’o was used to assign encoded enzymes to metabolic pathways based on the KOfam database (https://www.kegg.jp/kegg/ko.html). Since the genomes of the two novel isolates are nearly identical, the analysis was only performed for Strain 318^T^. The obtained results are in line with the heterotrophic lifestyle of the strain as was expected from a growth medium supporting heterotrophic growth. All enzymes required for a functional glycolysis (Embden-Meyerhof pathway), tricarboxylic acid cycle, pentose phosphate pathway and oxidative phosphorylation are present. The same is true for anabolic pathways yielding sugars (gluconeogenesis), amino acids, fatty acids, and ribonucleotides.

Apart from these expected results, the analysis also identified genes encoding subunits of an anoxygenic photosystem II (PufM, PufL). Since these proteins were also found in the closely related photoheterotroph *G. phototrophica*, the organization of the encoding genes in Strain 318^T^ was analysed in more detail. *G. phototrophica* harbours a genomic island of photosynthetic genes that was probably acquired via horizontal gene transfer^20^. Enzymes encoded by these genes are involved in bacteriochlorophyll biosynthesis (*bch* genes) and photosystem II reaction center assembly (*puh* and *puf* genes). The genomic island in *G. phototrophica* AP64^T^ is organized in two clusters with 16 and 14 genes, respectively, interrupted by six open reading frames unrelated to photosynthesis^20^. These two photosynthesis-related clusters are present in the genome of Strain 318^T^ in the identical gene order, with the sole difference that the second cluster is inverted and the clusters are separated by 39 open reading frames not related to photosynthesis. The genomic island is absent in the type strain of the more closely related strain *G. aurantiaca*. Hence, the two novel isolates are members of the PGB group together with *G. phototrophica* and *G. groenlandica*.

In addition, we looked deeper into carbohydrate-active enzymes (CAZymes) and secondary metabolite-associated biosynthetic gene clusters (BGCs) (Table 1). Numbers of CAZyme-encoding genes range from 19 to 135 and the ranking regarding genome size is the same as the ranking regarding CAZyme numbers. The most common CAZyme classes in the phylum are glycosyltransferases and glycoside hydrolases, with the type strain of *G. kalamazoonensis* being particularly enriched in enzymes of the latter class.

**Table 1:** Phenotypic and genomic features of the novel isolates in comparison to the type strains of closely related species.

Characteristic	Strain 318^ T^	Strain 138	*Gemmatimonas aurantiaca* T-27^ T ^	*Gemmatirosa kalamazoonensis* KBS708^T^	*Roseisolibacter agri* AW1220^T^	*Longimicrobium terrae* CB-286315^ T^
Sampled material	Freshwater sponge *Spongilla lacustris*	Freshwater sponge *Spongilla lacustris*	Anaerobic–aerobic sequential batch reactor for wastewater treatment	Organically-managed agricultural soil	Agricultural floodplain soil	Mediterranean forest soil
Isolation location (country)	Germany	Germany	Japan	USA	Namibia	Spain
Phenotypic features						
Size length (µm)	2.0 ± 0.6	2.0 ± 0.4	2.5–3.2	1.0–16.0	0.6–16.0	1.3–15.0 (up to 40.0)
Size width (µm)	0.50 ± 0.05	0.51 ± 0.04	0.7	0.5–0.7	0.4–0.5	0.4–0.6
Shape	short to long rod-shaped	short to long rod-shaped	Rod-shaped	Long and irregular rod-shaped	Long rod-shaped	Long and irregular rod-shaped
Aggregates	No	No	No	Yes	No	Yes
Colony colour	Light pink to pale salmon-colored	Light pink to pale salmon-colored	Faintly orange to pink	Light to dark pink	Whitish pink	Pale salmon
Division	Binary fission	Binary fission	Binary fission (sometimes budding forms)*	Binary fission (sometimes budding forms)*	Binary fission and/or budding*	Binary fission
Temperature range (optimum) (°C)	18–37 (28–30)	18–37 (28–30)	25–35 (30)	18–37 (33)	15–36 (20–25)	10–33 (25–28)
pH range (optimum)	7.0–9.0 (7.5)	7.0–8.0 (7.0)	6.5–9.5 (7.0)	5.0–7.0 (6.0)	6.4–8.4 (7.0–7.5)	6.0–9.0 (7.0–7.5)
Relation to oxygen	Aerobic	Aerobic	Aerobic	Microaerophilic to aerobic	Microaerophilic to aerobic	Microaerophilic to aerobic
Doubling time (h)	9	n.d.	12	n.d.	25	14
Genomic features						
Genome size (bp)	3,257,781	3,260,361	4,636,964	7,479,215	5,928,727	6,784,420
Plasmids	No	No	No	Putative extrachromosomal element	n.d.	n.d.
DNA G + C content (%)	68.7	68.7	64.2	72.6	73.9	69.5
Coding density (%)	93.3	93.2	93.0	91.8	90.0	83.1
Total genes	2967	2969	3998	6447	5074	5641
Genes/Mb	911	911	862	862	856	831
Protein-coding genes	2907	2909	3936	6323	5005	5502
Protein-coding genes/Mb	892	892	849	845	844	811
Hypothetical proteins	631	631	804	1499	3041	1661
Hypothetical proteins (%)	22	22	20	24	61	30
tRNA genes	46	46	46	50	64	60
rRNA genes (5S,16S,23S)	1,1,1	1,1,1	1,1,1	2,2,2	2,1,1	1,1,1
Carbohydrate-active enzymes						
Glycosyltransferases	16	16	20	33	26	24
Glycoside hydrolases	3	3	6	85	27	35
Carbohydrate esterases	0	0	1	8	0	0
Carbohydrate-binding modules	0	0	1	9	6	5
Biosynthetic gene clusters						
Terpenoid	2	2	2	2	2	1
Lanthipeptide	0	0	1	0	0	4
NRPS	0	0	1	1	0	4
Mixed type I PKS-NRPS	0	0	0	0	0	4
RRE-containing cluster	0	0	0	0	0	2

*Cell division by budding has been proposed in the original species descriptions, but our work suggests that the spherical appendages are not a division phenotype; n.d.: not determined/detected.

According to the antiSMASH results, members of the family *Gemmatimonadaceae* harbour between 2 and 4 BGCs independent of their genome size. The type strain of *L. terrae* (that belongs to the distinct family *Longimicrobiaceae*) harbours 15 BGCs although the genome size is only ranked second. The low numbers of BGCs obtained for the family *Gemmatimonadaceae* should not be overinterpreted, in particular when considering that the algorithm behind antiSMASH is trained with BGCs identified in other phyla that include well-characterized talented producers of bioactive compounds. It appears unlikely that the 7.5 Mb genome of the type strain of *G. kalamazoonensis* harbours less BGCs than the type strain of *G. aurantiaca* T27^T^, which has a 4.6 Mb genome. Terpenoid-synthesizing clusters are likely involved in the biosynthesis of carotenoid pigments. The carotenoid gemmatoxanthin was identified in *G. phototrophica* TET16^T^^21^, while *G. aurantiaca* T-27^T^ was shown to produce the glycosylated carotenoids oscillol-2,2′-di-rhamnoside and deoxyoscillol-2-rhamnoside^47^.

### The novel isolates are mesophilic and neutrophilic heterotrophs that do not show antimicrobial activity under laboratory-scale cultivation conditions

As a next step, we wanted to characterize the growth requirements and growth characteristics and investigate if the isolates can produce antimicrobial compounds beyond the ones included in current bioinformatic analysis pipelines. Both isolates are mesophilic (18–37 °C, optimum: 28–30 °C) and neutrophilic (pH 7.0–9.0, optimum: 7.0–7.5) (Table 1, Fig. S1). The temperature and pH preferences are comparable to the type strains of related species, except for *R. agri* that showed optimal growth at slightly lower temperatures (20–25 °C) and *G. kalamazoonensis* that preferred slightly more acidic conditions (pH 6.0). With a generation time of 9 h, growth of Strain 318^T^ is faster than that of other *Gemmatimonadota* strains for which the maximal growth rate has been determined. However, these values are difficult to compare since the used cultivation media differ. Supplementation of vitamins is not required for growth of the two novel strains in the tested medium. Colonies of Strain 318^T^ and Strain 138 have a light pink to pale salmon colour. The pigmentation is likely related to carotenoid formation already observed in other members of the phylum, fitting to the predictions based on the genome^48^. Extracts of the supernatant of a Strain 318^T^ culture did not show antimicrobial activity against a *tolC*-deficient *E. coli* strain (Gram-negative) and *B. subtilis* (Gram-positive, Fig. S2).

### *Gemmatimonadota* members divide symmetrically or asymmetrically likely employing FtsZ-based binary fission

**Figure 5:**
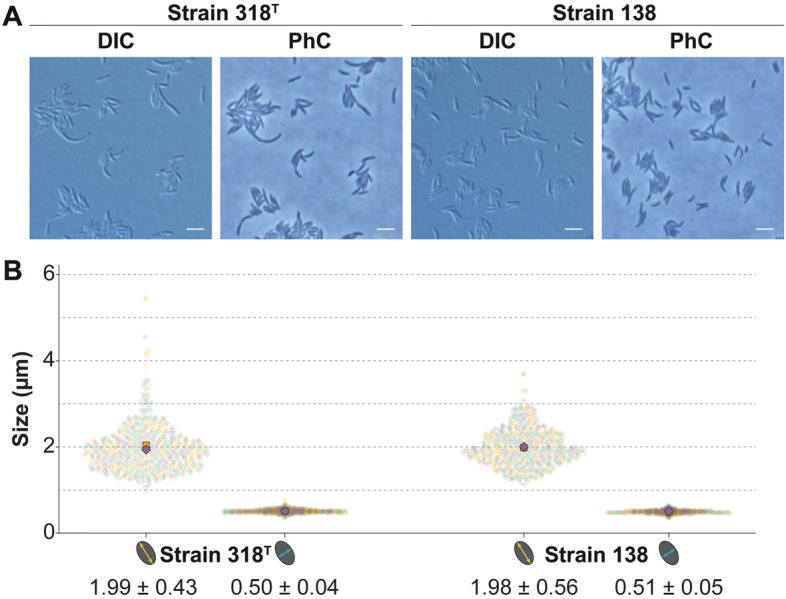
Light microscopy analysis shows that both strains have rod-shaped cells of varying length. (**A**) Differential interference contrast (DIC) and phase contrast (PhC) images of both strains show that these bacteria are rod-shaped. (**B**) Cell size determination of 150 cells per strain in biological triplicates shown in superplots, where each measurement is represented by a single dot and each replicate by a different colour and shape. Larger data points indicate the average per replicate. Scale bars represent 2 µm.

As we embarked on our journey to gain understanding of the diversity in bacterial cell biology, we studied the two novel isolates via extensive light microscopy experiments followed by quantitative image analysis, as well as electron microscopy. Cells of Strain 318^T^ and Strain 138 are rod-shaped and vary between 1.0 and 5.5 µm in size (Fig. 5A and Table 1). Their average cell length and width is 2.0 µm and 0.5 µm, respectively (Fig. 5B). Both strains can form loose and shapeless macroscopic aggregates in liquid medium but no microscopic aggregates, although occasionally, elongated cells stick together preventing the reliable use of automated image analysis software (Fig. 5B).

**Figure 6:**
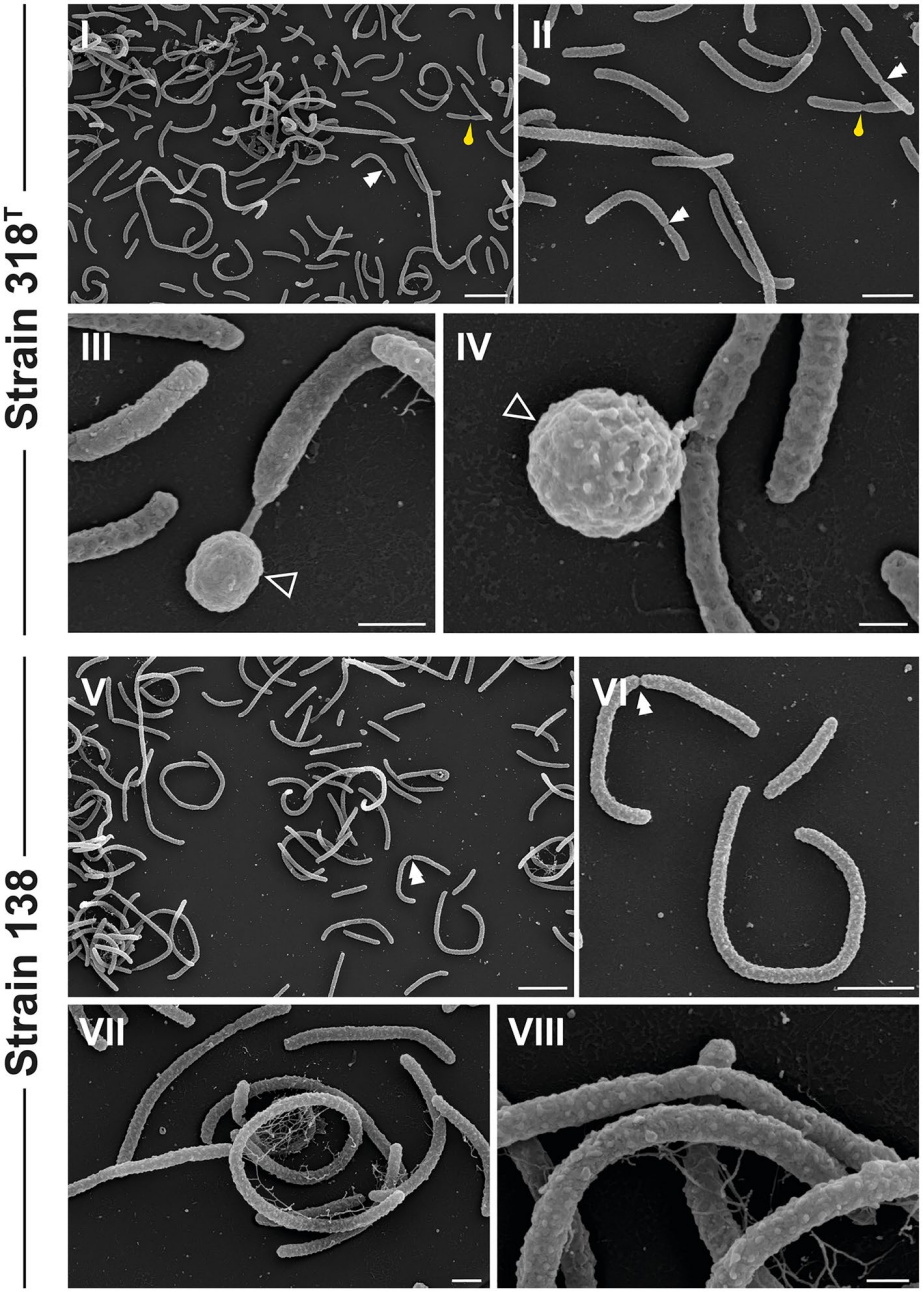
SEM images show that cells of both strains possess spherical appendages and display symmetric and asymmetric binary fission. Spherical appendages are indicated by non-filled triangles. Symmetrically dividing cells are indicated by yellow pins while asymmetrical division is indicated by white double arrow heads. Scale bars: 2 µm (I, II, V, VI), 0.2 µm (III, IV), 0.4 µm (VII, VIII). Images II and VI are cropped sections from image I and V, respectively.

All previously described members of the phylum *Gemmatimonadota* were reported to divide via binary fission and budding under laboratory conditions (with the exception of *L. terrae*, for which budding was not observed) (Table 1)^12–17^. SEM images of the type strains of *G. phototrophica* and *G. kalamazoonensis* provided a snapshot of cells dividing via a process that the authors termed ternary fission, a mode of cell division in which a second daughter cell emerges perpendicular to the division plane simultaneous to the regular binary fission event^14,17^. The formation of spherical appendages by (elongated) rod-shaped cells, interpreted as buds by the community till now^12–15,17^, was also observed for the here characterized isolates (Fig. 6).

We thus wanted to better understand the cell division process in the phylum, in which cells are apparently capable of symmetric and asymmetric binary and ternary fission as well as budding. We therefore followed cell division events via time-lapse microscopy. As this technique can show division events happening in real time, such experiments are crucial to properly understand cell division and had not been previously performed on strains belonging to this phylum. Specifically, we were interested to see if the spherical structures described as buds indeed pinch off from the mother cell and become viable daughter cells that start propagating themselves.

**Figure 7:**
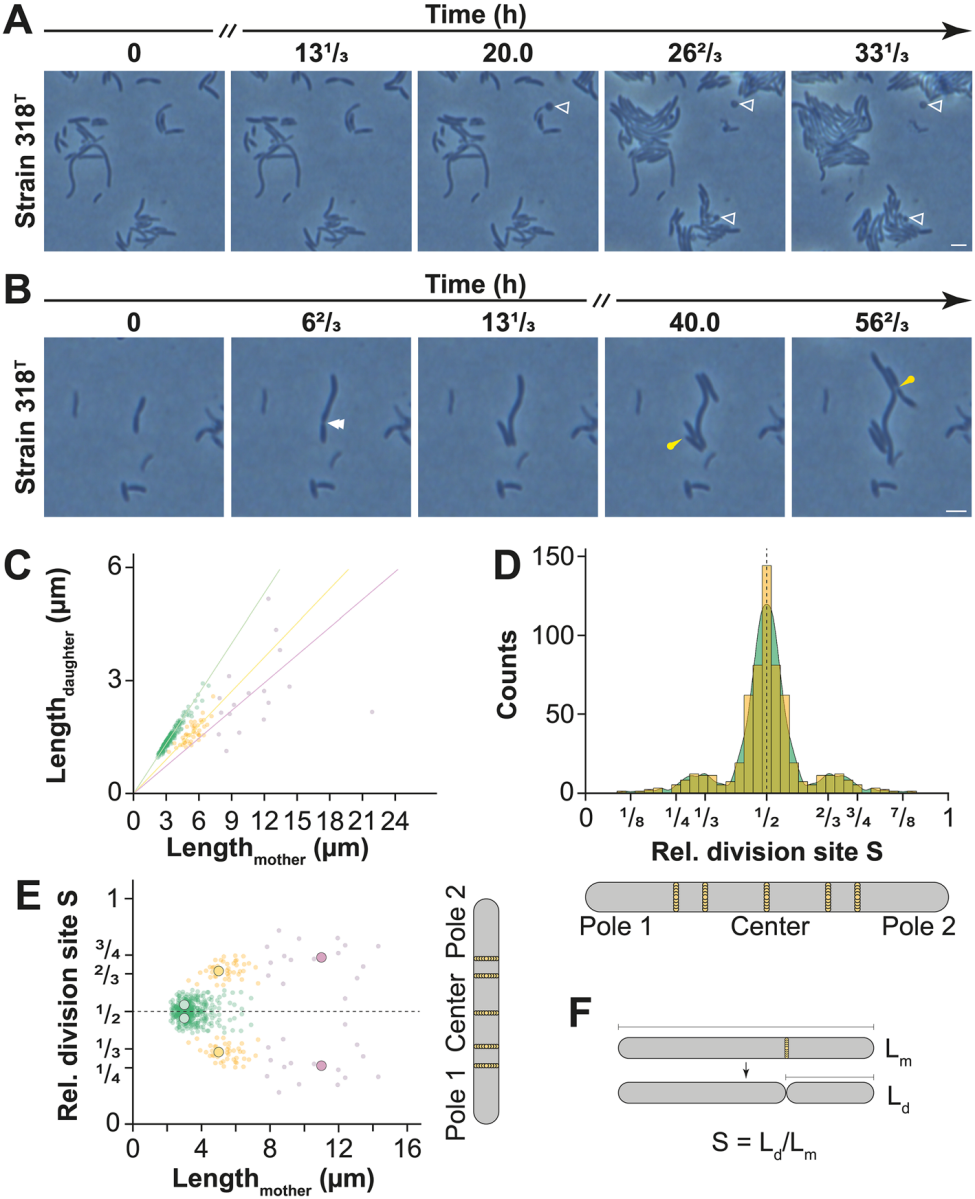
Time-lapse microscopy and analysis of division sites show that cells do not divide by budding but by consecutive fission events at multiple different planes. (**A** and **B**) Prolonged time-lapse experiments show (**A**) the formation of spherical appendages (white empty triangles) that stay inert as well as (**B**) cell division by asymmetric (white double arrow heads) and symmetric (yellow pins) binary fission. (**C**) Visualization of mother vs. daughter cell lengths (300 cells) shows that most cells divide at mid-cell and form a daughter cell similar in length to its mother cell (green line). However, a minor fraction of the population is able to produce daughter cells smaller in size than their mother and therefore divide asymmetrically at either 1/3rd (yellow line) or 1/4th (purple line). (**D**) Analysis of the division plane position based on 300 cells visualized as a histogram (yellow) and a density curve (green); indicating that the majority of cells divides via symmetric binary fission at mid-cell, whereas minor fractions divide at 1/3rd or 2/3rd and even less cells at 1/4th ad 3/4th of the cell length. (**E**) Visualization of the relative division site S with respect to cell length. Short cells divide symmetrically at 1/2, longer cells at 1/3rd or 2/3rd and even longer cells at 1/4th or 3/4th. (**F**) Calculation used to determine the relative division site S. Each dot represents the measurement of a single cell and larger dots represent the average value. Scale bars represent 2 µm, dashed lines indicate the plane where the data was mirrored to indicate potential divisions on both cell poles.

**Figure 8:**
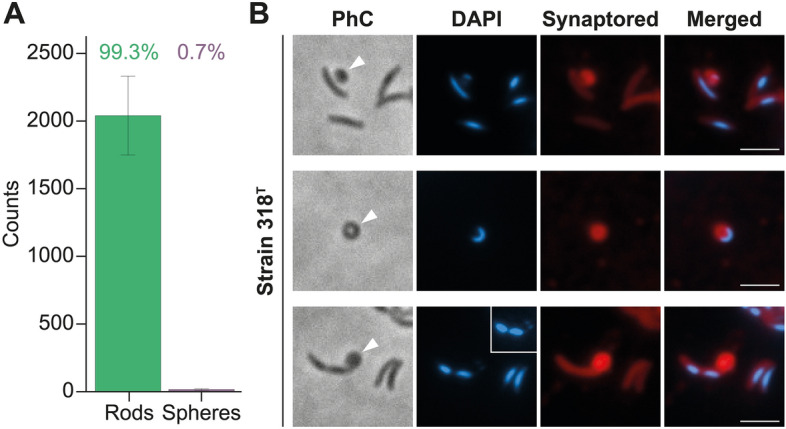
Microscopic analyses of spherical structures formed by Strain 318^T^. (**A**) The majority of cells of Strain 318^T^ are rod-shaped while the spherical structures can only be found in rare cases (0.7%). (**B**) Rod-shaped cells and spherical structures (white arrow head) contain DNA (DAPI) and appear to be enclosed by a membrane (Synaptored). The inlay contains an increased fluorescence intensity to display the weak DAPI signal in the spherical structure. Scale bars represent 2 µm.

Long-term time-lapse microscopy experiments showed that these spherical structures did not grow and did not pinch off from the mother cell (Fig. 7A, Movie S1). In fact, they did not change their shape at all, or in rare occasions disappeared through apparent lysis. Compared to rod-shaped cells (making up 99.3% of the analysed set of more than 18,000 cells, Fig. 8A) the spherical structures could rarely be found (0.7%, Fig. 8A) and thus represent an exceptional feature rather than a typical mode of reproduction.

Having established that the spherical structures do not represent viable budding daughter cells, we looked for indications what the nature of these structures could be. We wondered if spherical structures of Strain 318^T^ contain DNA and a membrane and to this end stained cells of this strain with DAPI and Synaptored resulting in the identification of DNA and a membrane, respectively (Fig. 8B). We occasionally observed that entire rod-shaped cells were sucked into the spherical structure (Movie S1), potentially explaining why these spherical structures contain DNA. Such a behaviour and the lysis of the spherical structures could resemble cells falling victim to phage infections, however, we could not identify a complete and conserved set of phage genes in the genomes of Strain 318^T^, Strain 138 or any other of the “budding” members in the family. Based on the morphology, an alternative explanation could be that the structures are (exo)spores. In this case, one would expect sporulation genes present in the genome. According to the annotation of the genome of Strain 318^T^ we could identify a partial set of potential *spo* genes, namely *spoIID/lytB*, *spoIIE*, *SPOR*, and *abrB/mazE/spoVT*. However, a *spo0A* gene encoding the master regulator of spore formation could not be identified. Additional important genes like *spoIIAB*, *spoIII*, *spoIV*, *spoV*, *spoVI* were not detected^49^. Phenotypically, one would further expect outgrowth of spores under favourable conditions (as in principle present under the microscope during the time-lapse experiment performed in the presence of growth medium) as opposed to lysis, as exospores are by definition very sturdy. In summary, while these spherical structures contain DNA and possess a kind of cell membrane, they only appear in rare occasions and under laboratory conditions did not show any form of growth. Supported by the absence of several genes required for spore formation, we therefore conclude that they do not play a role in the regular cell division process. The actual function of the spherical appendages (if present) remains enigmatic and requires further attention in future studies.

Having shown that cells of the novel gemmatimonadotal species do not divide via budding, we decided to study cell division more extensively. As light- and electron micrographs (Fig. 6) suggested that some cells divide at mid-cell and some divide in an asymmetric fashion, we decided to follow cell division events during time-lapse microscopy (Fig. 7B, Movie S2) and determine the relative division site (S) via quantitative image analysis (Fig. 7C-E). These analyses show that under laboratory conditions binary fission is the cell division mechanism in this species. However, longer cells (> 5 µm) often showed asymmetric binary fission, with the relative division site S located at or close to 1/3rd (or 2/3rd) and in even longer cells (approximately > 8 µm) close to 1/4th (or 3/4th) of the cell length (Fig. 7D-E).

With the lack of genetic tools for this phylum, it is challenging to get to a deeper understanding of the mechanism governing (asymmetric) binary fission in *Gemmatimonadota*. However, first indications could be obtained via a genome-based analysis (Tables S5 and S6). The in silico analysis showed the presence of the canonical *fts* gene set encoding for what appears to be a FtsZ-based divisome complex that is responsible for binary fission in most bacteria. In contrast to the divisome machinery, division site placement uses different mechanisms and proteins in different lineages. The known mechanisms include the Min system (based on MinCDE) present in for instance *E. coli *^50^, MipZ in *Caulobacter crescentus *^51^, PomZ in *Myxococcus xanthus *^52^*,* MapZ in *Streptococcus pneumoniae *^53^ and SsgAB in *Streptomyces coelicolor *^54^. The gemmatimonadotal genomes seem not to encode any of these proteins and therefore might use a different mechanism to determine the division site. Putative candidates for involvement in this mechanism might be the ParA-like proteins encoded outside of the *parAB* operon (involved in chromosome partitioning), as ParA-like proteins (incl. MinD, MipZ and PomZ) take on many roles in spatiotemporal organisation in bacterial cells^55^. The genome analysis indeed showed the presence of additional ParA-like proteins in multiple species of the phylum *Gemmatimonadota*, which would be excellent targets for in-depth studies. Unravelling the mechanism responsible for division site placement is of extra relevance as it leads to a different outcome as compared to the Min system^56^ and might thus be fundamentally different. The difference comes across the distribution of cell lengths, as well as where extraordinarily long cells divide. While elongated cells using the Min system for division site placement (for instance *E. coli*) seem to choose a location at even fractions like 1/4th or 3/4th (and for longer cells 1/6th, 3/6th or 5/6th and 1/8th, 3/8th, 5/8th, or 7/8th) of the cell length^56^, long cells of Strain 318^T^ also divide at odd fractions (preferentially 1/3rd or 2/3rd). Taken together, studying such non-model bacteria can lead to interesting avenues. The obtained insights can add or challenge fundamental concepts helping to understand the diversity in systems of the bacterial cell biology.

## Conclusion

The here employed untargeted cultivation strategy combined a low-nutrient cultivation medium with long incubation times and empirically determined inoculation volumes. It turned out to be suitable for the isolation of uncharacterized bacterial strains from environmental sponge samples. Based on the phylogenetic distance and supported by phenotypic differences, the two isolates should be delineated from the described genera in the family *Gemmatimonadaceae.* Cells of the novel isolates divide symmetrically or asymmetrically, likely employing a binary fission mechanism involving canonical bacterial division proteins. Like most of the other members of the phylum, cells can form spherical appendages emerging from the cell poles. However, based on our data, these structures are unrelated to cell division and their exact function remains enigmatic.

### Description of *Pseudogemmatithrix* gen. nov.

Pseu.do.gem.ma’ti.thrix. Gr. adj. *pseudes*, false, mendacious; L. masc. part. adj. *gemmatus*, provided with buds; Gr. fem. n. *thrix*, hair, thread; N.L. fem. n. *Pseudo*g*emmatithrix*, a false bud-forming thread.

Gram-negative. Aerobic heterotrophs with a mesophilic and neutrophilic growth profile. Cells are short to long rods that divide by binary fission. Formation of spherical appendages is probably unrelated to cell division. The DNA G + C content of its members is around 69%. The genus belongs to the family *Gemmatimonadaceae*, order *Gemmatimonadales,* class *Gemmatimonadia,* phylum *Gemmatimonadota*. The type species of this genus is *Pseudo*g*emmatithrix spongiicola*.

### Description of *Pseudogemmatithrix spongiicola* sp. nov.

spon.gi.i’co.la. L. fem. n. *spongia*, sponge; L. masc./fem. n. suff. *-cola*, inhabitant, dweller; from L. masc./fem. n. *incola*, dweller; N.L. masc./fem. n. *spongiicola*, sponge inhabitant.

Rod-shaped and pale pink to salmon-pigmented cells with an average size of 2.0 × 0.5 μm. Longer cells were observed at the late exponential phase. Growth is observed over a range of 18–37 °C (optimum 28–30 °C) and at pH 7.0–9.0 (optimum 7.5). Vitamins are not required for growth. Potentially photoheterotrophic. The type strain has a genome size of 3.26 Mb and a DNA G + C content of 68.7%. The type strain is Strain 318^ T^ (= DSM 109487^ T^ = CECT 9875^ T^ = LMG 31380^ T^ = VKM B-3446^ T^). It was isolated from a specimen of the freshwater sponge *Spongilla lacustris* sampled in the Salzgitter Lake in the Northern Germany in August 2016. An additional strain belonging to this species is Strain 138 (= DSM 109757 = CECT 9874).

### Supplementary Information

Supplementary Information 1.

Supplementary Video 1.

Supplementary Video 2.

## Data Availability

The 16S rRNA gene sequence of the two isolates is available from GenBank under the accession numbers OR304275 (Strain 138) and OR336385 (Strain 318^T^). The genome sequence can be found under the accession numbers CP130612 (Strain 138) and CP130613 (Strain 318^T^).
