# Supplementary material for: An untargeted cultivation approach revealed Pseudogemmatithrix spongiicola gen. nov., sp. nov., and sheds light on the gemmatimonadotal mode of cell division: binary fission
Source: Sci Rep. 2024 Jul 21;14:16764. doi: 10.1038/s41598-024-67408-9 (PMC11271474; doi:10.1038/s41598-024-67408-9)
Supplement: Supplementary file 1 — Supplementary Information 1. [file 41598_2024_67408_MOESM1_ESM.pdf]

Supplementary Material

An untargeted cultivation approach revealed *Pseudogemmatithrix spongiicola* gen. nov., sp. nov., and sheds light on the gemmatimonadotal mode of cell division: binary fission

Tom Haufschild[1,#], Nicolai Kallscheuer[1,#], Jonathan Hammer[1], Timo Kohn[2], Moses Kabuu[1], Mareike Jogler[1], Nicole Wohlfarth[1], Manfred Rohde[3], Muriel C.F. van Teeseling[1], Christian Jogler[1,4]*

[1] Department of Microbial Interactions, Institute of Microbiology, Friedrich Schiller University, Jena, Germany

[2] Leibniz Institute DSMZ, Brunswick, Germany

[3] Central Facility for Microscopy, Helmholtz Centre for Infection Research, Brunswick, Germany

[4] Cluster of Excellence Balance of the Microverse, Friedrich Schiller University, Jena, Germany

[#] authors contributed equally to this work

* corresponding author: christian.jogler@uni-jena.de

Keywords: aquatic microbiology, freshwater sponge, *Gemmatimonas*, cell division, binary fission, budding

Running title: A novel genus in the phylum *Gemmatimonadota*

Supplementary Tables

Table S1. 16S rRNA gene similarity matrix. Similarity values are provided in %.

	Gemmatirosa kalamazoonensis	*Roseisolibacter agri*	*Gemmatimonas aurantiaca*	*Gemmatimonas groenlandica*	*Gemmatimonas phototrophica*	*Longimicrobium terrae*	Strain 138	Strain 318
Gemmatirosa kalamazoonensis	100.00	91.98	90.00	91.25	90.33	84.09	89.54	89.54
Roseisolibacter agri	91.98	100.00	90.31	91.38	90.17	83.36	87.90	87.90
Gemmatimonas aurantiaca	90.00	90.31	100.00	96.32	95.99	84.35	91.06	91.06
Gemmatimonas groenlandica	91.25	91.38	96.32	100.00	95.93	84.35	90.93	90.93
Gemmatimonas phototrophica	90.33	90.17	95.99	95.93	100.00	83.69	91.65	91.65
Longimicrobium terrae	84.09	83.36	84.35	84.35	83.69	100.00	82.76	82.76
Strain 138	89.54	87.90	91.06	90.93	91.65	82.76	100.00	100.00
Strain 318	89.54	87.90	91.06	90.93	91.65	82.76	100.00	100.00

Table S2. Average amino acid (AAI) similarity matrix. Similarity values are provided in %.

	Gemmatimonas aurantiaca	*Gemmatimonas groenlandica*	*Gemmatimonas phototrophica*	*Gemmatirosa kalamazoonensis*	*Longimicrobium terrae*	*Roseisolibacter agri*	Strain 138	Strain 318
Gemmatimonas aurantiaca	100.00	69.74	72.15	55.36	46.43	56.26	54.64	54.65
Gemmatimonas groenlandica	69.74	100.00	70.96	55.66	46.44	56.61	55.13	55.12
Gemmatimonas phototrophica	72.15	70.96	100.00	55.42	46.15	56.43	55.34	55.34
Gemmatirosa kalamazoonensis	55.36	55.66	55.42	100.00	48.60	64.58	54.80	54.82
Longimicrobium terrae	46.43	46.44	46.15	48.60	100.00	48.99	46.49	46.49
Roseisolibacter agri	56.26	56.61	56.43	64.58	48.99	100.00	55.72	55.71
Strain 138	54.64	55.13	55.34	54.80	46.49	55.72	100.00	100.00
Strain 318	54.65	55.12	55.34	54.82	46.49	55.71	100.00	100.00

Table S3. Average nucleotide identity (ANI) matrix. Similarity values are provided in %.

	Gemmatimonas aurantiaca	*Gemmatimonas groenlandica*	*Gemmatimonas phototrophica*	*Gemmatirosa kalamazoonensis*	*Longimicrobium terrae*	*Roseisolibacter agri*	Strain 138	Strain 318
Gemmatimonas aurantiaca	100.00	78.35	78.61	76.04	74.31	75.93	76.42	76.41
Gemmatimonas groenlandica	78.35	100.00	78.01	75.37	73.09	75.85	75.75	75.74
Gemmatimonas phototrophica	78.61	78.01	100.00	76.00	73.99	76.43	76.10	76.06
Gemmatirosa kalamazoonensis	76.04	75.37	76.00	100.00	74.11	78.88	76.43	76.44
Longimicrobium terrae	74.31	73.09	73.99	74.11	100.00	74.15	73.88	73.90
Roseisolibacter agri	75.93	75.85	76.43	78.88	74.15	100.00	76.77	76.79
Strain 138	76.42	75.75	76.10	76.43	73.88	76.77	100.00	100.00
Strain 318	76.41	75.74	76.06	76.44	73.90	76.79	100.00	100.00

Table S4. Percentage of conserved proteins (POCP) matrix. Similarity values are provided in %.

	Gemmatimonas aurantiaca	*Gemmatimonas groenlandica*	*Gemmatimonas phototrophica*	*Gemmatirosa kalamazoonensis*	*Longimicrobium terrae*	*Roseisolibacter agri*	Strain 138	Strain 318
Gemmatimonas aurantiaca	100.00	65.73	67.95	43.90	29.53	48.89	47.37	47.38
Gemmatimonas groenlandica	65.73	100.00	74.77	45.87	29.64	50.47	48.21	48.24
Gemmatimonas phototrophica	67.95	74.77	100.00	44.09	28.56	48.98	50.13	50.18
Gemmatirosa kalamazoonensis	43.90	45.87	44.09	100.00	33.35	61.70	37.79	37.82
Longimicrobium terrae	29.53	29.64	28.56	33.35	100.00	34.76	28.09	28.10
Roseisolibacter agri	48.89	50.47	48.98	61.70	34.76	99.98	43.18	43.21
Strain 138	47.37	48.21	50.13	37.79	28.09	43.18	100.00	99.88
Strain 318	47.38	48.24	50.18	37.82	28.10	43.21	99.88	100.00

Table S5. Presence of genes involved in cell division and chromosome segregation. n: gene not found, y: gene found via KEGG Kofams, (y): gene not found via KEGG Kofams, but identified via manual Blast analysis, number represents the number of genes found via KEGG Kofams

Gene	Annotation	Kofam	Strain 138	Strain 318	*Gemmatimonas aurantiaca*	*Gemmatimonas groenlandica*	*Gemmatimonas phototrophica*	*Gemmatirosa kalamazoonensis*	*Longimicrobium terrae*	*Roseisolibacter agri*
**Divisome proteins**										
ftsA	cell division protein FtsA	K03590	**y**	**y**	**y**	**y**	**y**	**y**	**y**	**y**
ftsB	cell division protein FtsB	K05589	**y**	**y**	**y**	**y**	**y**	**y**	**y**	**y**
ftsE	cell division transport system ATP-binding protein	K09812	**y**	**y**	**y**	**y**	**y**	**y**	**y**	**y**
ftsI	cell division protein FtsI	K03587	**y**	**y**	**y**	**y**	**y**	**y**	**y**	**y**
ftsK	spoIIIE; DNA segregation ATPase FtsK/SpoIIIE	K03466	**y**	**y**	**y**	**y**	**y**	**y**	**y**	**y**
ftsL	cell division protein FtsL	K03586	**y**	**y**	**y**	**y**	**y**	**y**	**y**	**y**
ftsN	cell division protein FtsN	K03591	**(y)**	**(y)**	**(y)**	**(y)**	**(y)**	**(y)**	**(y)**	**y**
ftsQ	cell division protein FtsQ	K03589	**y**	**y**	**y**	**y**	**y**	**y**	**y**	**y**
ftsW	spoVE; cell division protein FtsW	K03588	**y**	**y**	**y**	**y**	**y**	**y**	**y**	**y**
ftsX	cell division transport system permease protein	K09811	**y**	**y**	**y**	**y**	**y**	**y**	**y**	**y**
ftsZ	cell division protein FtsZ	K03531	**y**	**y**	**y**	**y**	**y**	**y**	**y**	**y**
zipA	cell division protein ZipA	K03528	**n**	**n**	**n**	**n**	**n**	**n**	**n**	**n**
zapA	cell division protein ZapA	K09888	**y**	**y**	**y**	**y**	**y**	**y**	**y**	**y**
zapB	cell division protein ZapB	K09892	**n**	**n**	**n**	**n**	**n**	**n**	**n**	**n**
zapC	cell division protein ZapC	K18657	**n**	**n**	**n**	**n**	**n**	**n**	**n**	**n**
zapD	cell division protein ZapD	K18778	**n**	**n**	**n**	**n**	**n**	**n**	**n**	**n**
zapE	cell division protein ZapE	K06916	**n**	**n**	**n**	**n**	**n**	**n**	**n**	**n**
ytfB	cell division protein YtfB	K07269	**n**	**n**	**n**	**n**	**n**	**n**	**n**	**n**
**Division site placement**										
minC	septum site-determining protein MinC	K03610	**n**	**n**	**n**	**n**	**n**	**n**	**n**	**n**
minD	septum site-determining protein MinD	K03609	**n**	**n**	**n**	**n**	**n**	**n**	**n**	**n**
minE	cell division topological specificity factor	K03608	**n**	**n**	**n**	**n**	**n**	**n**	**n**	**n**
parA, soj	chromosome partitioning protein	K03496	**2**	**2**	**2**	**2**	**2**	**2**	**4**	**3**
parB, spo0J	ParB family transcriptional regulator	K03497	**y**	**y**	**y**	**y**	**y**	**y**	**y**	**y**
**Inhibitors of FtsZ assembly**										
ezrA	septation ring formation regulator	K06286	**n**	**n**	**n**	**n**	**n**	**n**	**n**	**n**
sulA	cell division inhibitor SulA	K13053	**n**	**n**	**n**	**n**	**n**	**n**	**n**	**n**
sepF	cell division inhibitor SepF	K09772	**n**	**n**	**n**	**n**	**n**	**n**	**n**	**n**

Table S6. Presence of genes involved peptidoglycan biosynthesis and additional biological processes. n: gene not found, y: gene found via KEGG Kofams, (y): gene not found via KEGG Kofams, but identified via manual Blast analysis, number represents the number of genes found via KEGG Kofams

Gene	Annotation	Kofam	Strain 138	Strain 318	*Gemmatimonas aurantiaca*	*Gemmatimonas groenlandica*	*Gemmatimonas phototrophica*	*Gemmatirosa kalamazoonensis*	*Longimicrobium terrae*	*Roseisolibacter agri*
**Peptidoglycan biosynthesis and remodeling**										
murA	UDP-N-acetylglucosamine 1-carboxyvinyltransferase	K00790	y	y	y	y	y	y	y	y
murB	UDP-N-acetylmuramate dehydrogenase	K00075	y	y	y	y	y	y	y	y
murC	UDP-N-acetylmuramate--alanine ligase	K01924	y	y	y	y	y	y	y	y
murD	UDP-N-acetylmuramoylalanine--D-glutamate ligase	K01925	y	y	y	y	y	y	y	y
murE	UDP-N-acetylmuramoyl-L-alanyl-D-glutamate--2,6-diaminopimelate ligase	K01928	y	y	y	y	y	y	y	y
murF	UDP-N-acetylmuramoyl-tripeptide--D-alanyl-D-alanine ligase	K01929	y	y	y	y	y	y	y	y
mraY	phospho-N-acetylmuramoyl-pentapeptide-transferase	K01000	y	y	y	y	y	y	y	y
murG	UDP-N-acetylglucosamine--N-acetylmuramyl-(pentapeptide) pyrophosphoryl-undecaprenol N-acetylglucosamine transferase	K02563	y	y	y	y	y	y	y	y
murJ	putative peptidoglycan lipid II flippase	K03980	y	y	y	y	y	y	y	y
ddlB	D-alanine-D-alanine ligase	K01921	y	y	y	y	y	y	y	y
mrdA	penicillin-binding protein 2	K05515	y	y	y	y	y	y	y	y
mraW	16S rRNA (cytosine1402-N4)-methyltransferase	K03438	y	y	y	y	y	y	y	y
amiABC	N-acetylmuramoyl-L-alanine amidase	K01448	y	y	y	y	y	y	y	y
envC	murein hydrolase activator	K22719	y	y	y	y	y	y	y	y
**Further genes of cell biological interest**										
seqA	negative modulator of initiation of replication	K03645	n	n	n	n	n	n	n	n
mreB	rod shape-determining protein MreB and related proteins	K03569	y	y	y	y	y	y	y	y
mreC	rod shape-determining protein MreC	K03570	y	y	y	y	y	y	y	y
mreD	rod shape-determining protein MreD	K03571	y	y	y	y	y	y	n	y
rodA	mrdB; rod shape determining protein RodA	K05837	y	y	y	y	y	y	y	y
rodZ	cytoskeleton protein RodZ	K15539	n	n	n	n	n	n	n	n
parC	topoisomerase IV subunit A	K02621	n	n	n	n	n	n	n	n
parE	topoisomerase IV subunit B	K02622	n	n	n	n	n	n	n	n
divIVA	cell division initiation protein	K04074	y	y	y	y	y	y	y	y
popZ	cell pole-organizing protein PopZ	K09991	n	n	n	n	n	n	n	n
gidA, mnmG	tRNA uridine 5-carboxymethylaminomethyl modification enzyme	K03495	y	y	y	y	y	y	y	y
gidB, rsmG	16S rRNA (guanine527-N7)-methyltransferase	K03501	n	n	n	n	n	n	n	n
trmFO, gid	methylenetetrahydrofolate--tRNA-(uracil-5-)-methyltransferase	K04094	y	y	y	y	y	y	y	y
xerC	integrase/recombinase XerC	K03733	y	y	y	y	y	y	y	y
xerD	integrase/recombinase XerD	K04763	y	y	y	y	y	y	y	y
racA	chromosome-anchoring protein RacA	K11686	n	n	n	n	n	n	n	n
yabN	tetrapyrrole methylase family protein / MazG family protein	K02499	n	n	n	n	n	n	n	n
mrp, NUBPL	ATP-binding protein involved in chromosome partitioning	K03593	y	y	y	y	y	y	y	y
engB	GTP-binding protein	K03978	y	y	y	y	y	y	y	y
whiA	cell division protein WhiA	K09762	n	n	n	n	n	n	n	n

Supplementary Figures

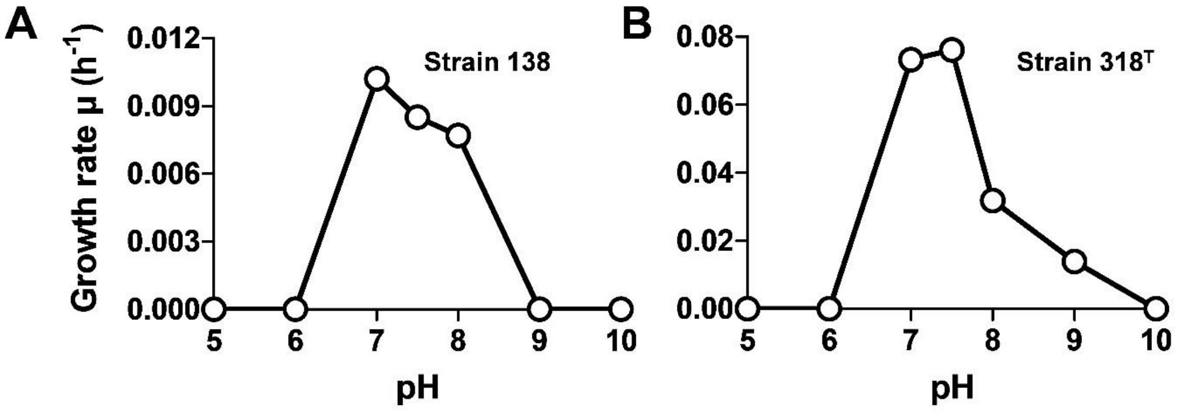

Figure S1. Determination of the pH optimum for growth. The maximal mean growth rates obtained during cultivation of the novel isolates with varying pH values from two biological replicates and two technical replicates are show.

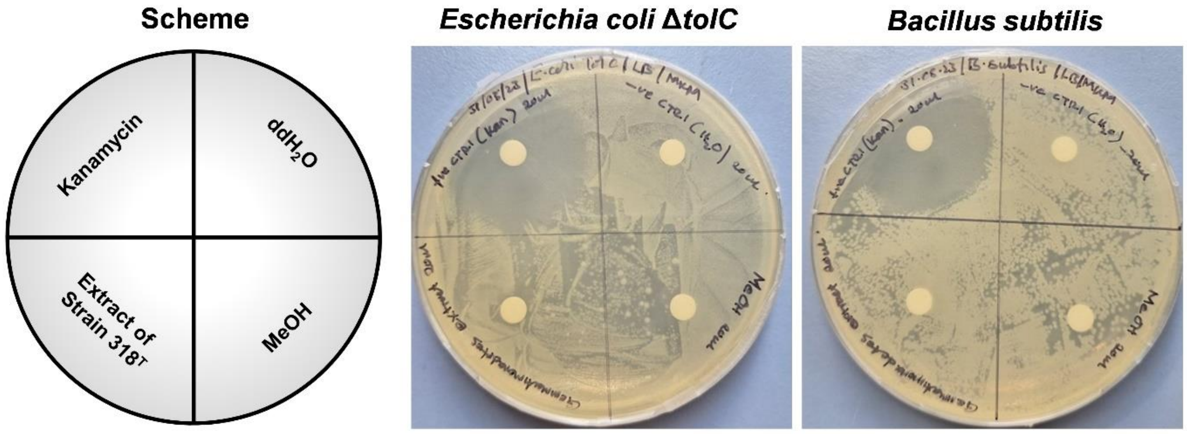

Figure S2. Antimicrobial activity assay. Ethyl acetate extracts of a culture of Strain 318^T were tested for antimicrobial activity against two indicator strains. Kanamycin was used a positive control and ddH$_2$O and MeOH served as negative controls.

Supplementary Movies

Movie S1. Formation of spherical appendages. Growth of Strain 318^T was investigated during a time-lapse microscopy experiment. Representative frames are shown in Figure 7A. Formed appendages either remained attached to the cell or incorporated the rod-shaped cell.

Movie S2. Cell division by binary fission. Growth of Strain 318^T was investigated during a time-lapse microscopy experiment. Representative frames are shown in Figure 7B. Cell division either took place in a symmetric or asymmetric manner.
